# HIV-1-Transmitted Drug Resistance and Transmission Clusters in Newly Diagnosed Patients in Portugal Between 2014 and 2019

**DOI:** 10.3389/fmicb.2022.823208

**Published:** 2022-04-25

**Authors:** Marta Pingarilho, Victor Pimentel, Mafalda N. S. Miranda, Ana Rita Silva, António Diniz, Bianca Branco Ascenção, Carmela Piñeiro, Carmo Koch, Catarina Rodrigues, Cátia Caldas, Célia Morais, Domitília Faria, Elisabete Gomes da Silva, Eugénio Teófilo, Fátima Monteiro, Fausto Roxo, Fernando Maltez, Fernando Rodrigues, Guilhermina Gaião, Helena Ramos, Inês Costa, Isabel Germano, Joana Simões, Joaquim Oliveira, José Ferreira, José Poças, José Saraiva da Cunha, Jorge Soares, Júlia Henriques, Kamal Mansinho, Liliana Pedro, Maria João Aleixo, Maria João Gonçalves, Maria José Manata, Margarida Mouro, Margarida Serrado, Micaela Caixeiro, Nuno Marques, Olga Costa, Patrícia Pacheco, Paula Proença, Paulo Rodrigues, Raquel Pinho, Raquel Tavares, Ricardo Correia de Abreu, Rita Côrte-Real, Rosário Serrão, Rui Sarmento e Castro, Sofia Nunes, Telo Faria, Teresa Baptista, Maria Rosário O. Martins, Perpétua Gomes, Luís Mendão, Daniel Simões, Ana Abecasis

**Affiliations:** ^1^Global Health and Tropical Medicine (GHTM), Instituto de Higiene e Medicina Tropical (IHMT), Universidade Nova de Lisboa (UNL), Lisbon, Portugal; ^2^Serviço de Infeciologia, Hospital Beatriz Ângelo, Loures, Portugal; ^3^Unidade de Imunodeficiência, Centro Hospitalar Universitário Lisboa Norte - HPV, Lisbon, Portugal; ^4^Serviço de Infeciologia, Centro Hospitalar de Setúbal, Setúbal, Portugal; ^5^Serviço de Doenças Infeciosas, Centro Hospitalar Universitário de São João, Porto, Portugal; ^6^Centro de Biologia Molecular, Serviço de Imunohemoterapia do Centro Hospitalar Universitário de São João, Porto, Portugal; ^7^Serviço de Medicina, Hospital de São José, Centro Hospitalar Universitário de Lisboa Central, Lisbon, Portugal; ^8^Serviço de Patologia Clínica, Centro Hospitalar e Universitário de Coimbra, Coimbra, Portugal; ^9^Serviço de Medicina, Hospital de Portimão, Centro Hospitalar Universitário do Algarve, Portimão, Portugal; ^10^Unidade Local de Saúde do Baixo Alentejo, Hospital José Joaquim Fernandes, Beja, Portugal; ^11^Serviço de Medicina, Hospital de Santo António dos Capuchos, Centro Hospitalar Universitário de Lisboa Central, Lisbon, Portugal; ^12^Hospital de Dia de Doenças Infeciosas, Hospital Distrital de Santarém, Santarém, Portugal; ^13^Serviço de Doenças Infeciosas, Hospital de Curry Cabral, Centro Hospitalar Universitário de Lisboa Central, Lisbon, Portugal; ^14^Serviço de Patologia Clínica, Hospital de Santa Maria, Centro Hospitalar Universitário de Lisboa Norte, Lisbon, Portugal; ^15^Serviço de Patologia Clínica, Centro Hospitalar do Porto, Porto, Portugal; ^16^Laboratório de Biologia Molecular (LMCBM, SPC, CHLO-HEM), Lisbon, Portugal; ^17^Serviço de Doenças, Centro Hospitalar e Universitário de Coimbra, Coimbra, Portugal; ^18^Serviço de Medicina, Hospital de Faro, Centro Hospitalar Universitário do Algarve, Faro, Portugal; ^19^Serviço de Doenças Infeciosas, Hospital de Egas Moniz, Centro Hospitalar de Lisboa Ocidental, Lisbon, Portugal; ^20^Serviço de Infeciologia, Hospital Garcia da Orta, Almada, Portugal; ^21^Serviço de Infeciologia, Centro Hospitalar do Porto, Porto, Portugal; ^22^Serviço de Infeciologia, Hospital de Aveiro, Centro Hospitalar Baixo Vouga, Aveiro, Portugal; ^23^Serviço de Infeciologia, Hospital Professor Doutor Fernando da Fonseca, Amadora, Portugal; ^24^Serviço de Patologia Clínica, Biologia Molecular, Centro Hospitalar Universitário de Lisboa Central, Lisbon, Portugal; ^25^Serviço de Infeciologia, Hospital de Faro, Centro Hospitalar Universitário do Algarve, Faro, Portugal; ^26^Serviço de Infeciologia, Unidade de Local de Saúde de Matosinhos, Hospital Pedro Hispano, Matosinhos, Portugal; ^27^Grupo de Ativistas em Tratamentos (GAT), Lisbon, Portugal; ^28^Centro de Investigação Interdisciplinar Egas Moniz (CiiEM), Instituto Universitário Egas Moniz, Costa da Caparica, Portugal

**Keywords:** HIV-1, TDR, transmission clusters, Portugal, newly infected patients

## Abstract

**Objective:**

To describe and analyze transmitted drug resistance (TDR) between 2014 and 2019 in newly infected patients with HIV-1 in Portugal and to characterize its transmission networks.

**Methods:**

Clinical, socioepidemiological, and risk behavior data were collected from 820 newly diagnosed patients in Portugal between September 2014 and December 2019. The sequences obtained from drug resistance testing were used for subtyping, TDR determination, and transmission cluster (TC) analyses.

**Results:**

In Portugal, the overall prevalence of TDR between 2014 and 2019 was 11.0%. TDR presented a decreasing trend from 16.7% in 2014 to 9.2% in 2016 (p_*for–trend*_ = 0.114). Multivariate analysis indicated that TDR was significantly associated with transmission route (MSM presented a lower probability of presenting TDR when compared to heterosexual contact) and with subtype (subtype C presented significantly more TDR when compared to subtype B). TC analysis corroborated that the heterosexual risk group presented a higher proportion of TDR in TCs when compared to MSMs. Among subtype A1, TDR reached 16.6% in heterosexuals, followed by 14.2% in patients infected with subtype B and 9.4% in patients infected with subtype G.

**Conclusion:**

Our molecular epidemiology approach indicates that the HIV-1 epidemic in Portugal is changing among risk group populations, with heterosexuals showing increasing levels of HIV-1 transmission and TDR. Prevention measures for this subpopulation should be reinforced.

## Introduction

The “Treatment for All” program was implemented in many countries with an aim to offer treatment and care to anyone diagnosed with HIV, regardless of the stage of infection (CD4 cell count). In Portugal, this program was implemented in 2015 [World Health Organiztion (WHO), 2021]. The widespread use and increased coverage of antiretroviral therapy (ART) have reduced the risk of HIV transmission, decreased HIV-related morbidity and mortality, and improved life quality. However, treatment scale-up can potentiate the risk for the development of antiretroviral (ARV) drug resistance, which can be transmitted to newly infected individuals (Palella et al., 1998; Lima et al., 1999; Clavel and Hance, 2004; Cohen et al., 2011). TDR in HIV-1-infected patients has become a major concern as it may lead to the failure of first-line ART. There are several studies indicating that the prevalence of TDR is largely variable in different settings and risk groups and that this could be related to the differences in the availability of treatment and levels of socioeconomic development (Pennings, 2013; Frentz et al., 2014; Yang et al., 2015). For example, TDR levels are highly discrepant when we compare Germany (18.4%) (van de Laar et al., 2019)[9], Belgium (15.7%) (van de Laar et al., 2019)[9], Hungary (7.1%) (van de Laar et al., 2019)[9], Netherlands (12.3%) (van de Laar et al., 2019)[9], Mozambique (14.0% in women) (Pina-Araujo et al., 2014)[10], Latin America (7.7%) (Avila-Rios et al., 2016)[11], and Washington DC (20.0%) (Aldous et al., 2017)[12]. Portugal, on the other hand, presented an overall TDR of 9.4% between 2001 and 2017, with a significantly increasing trend from 7.9% in 2003 to 13.1% in 2017 (Pingarilho et al., 2020). These results were obtained in a retrospective study of our study group and were based only on RegaDB, a laboratory database including clinical, demographic, and genomic data of patients followed up in hospitals located in the southern region of Portugal.

As the HIV epidemic continues to spread, it is very important to investigate the prevalence and transmission of TDR over the years in individual settings/locations. Moreover, the phylogenetic analysis provides insight into the HIV transmission clusters (TCs). The characterization of HIV-1 transmission clusters and associated TDR allows for targeted interventions to individuals at higher risk.

In this study, we aim to describe TDR between 2014 and 2019 in newly diagnosed patients with HIV-1 in Portugal, characterize the most prevalent drug resistance mutations, and identify predictors of TDR in Portugal. Furthermore, we aim to characterize HIV-1 transmission clusters involving these patients.

## Materials and Methods

### Study Population and Data Collection

The protocol was in accordance with the Declaration of Helsinki and approved by the Ethical Committee of all hospitals involved in the study.

Clinical, socioepidemiological, and risk behavior data were collected prospectively from 820 newly diagnosed patients from 17 hospitals located across the whole country from north to south of Portugal between September 2014 and December 2019. This sampling corresponds to a sampling rate of 17% of the total newly diagnosed cases in Portugal within these 5 years. The BEST HOPE database contains anonymized patients’ information, including demographic, clinical, behavioral, and genotype resistance data. The data of all the patients were generated in the context of routine clinical care.

### Drug Resistance Analyses and Subtyping

The genomic data included protease and reverse transcriptase sequences obtained through population sequencing performed at the molecular biology laboratories of different hospitals during daily care routine analysis. The genomic sequences were obtained for all the patients at the time of diagnosis, before starting ARV therapy. TDR was defined as the presence of one or more surveillance drug resistance mutations (SDRMs) according to the WHO 2009 surveillance list (Bennett et al., 2009)[14]. Nucleotide sequences were submitted to the Calibrated Population Resistance tool version 8.0. Clinical resistance to ARV drugs was inferred using the Stanford HIVdb v8.4. HIV-1 subtypes and circulating recombinant forms (CRFs) were determined as previously described (Pineda-Peña et al., 2013; Struck et al., 2014)[15,16]. Sequence alignments and associated metadata are available from the authors upon request.

### Late Presenters and Late Presenters With Advanced Disease

According to the European Late Presenter Consensus working group, late presenters (LP) were defined as a CD4 count lower than 350 cells/μL at the time of diagnosis or present with an AIDS-defining event at diagnosis, regardless of the CD4 cell count. A subgroup of late presenters, called late presenters with advanced disease (LPAD), were characterized by presenting a CD4 count lower than 200 cells/μL or an AIDS-defining event, regardless of the CD4 cell count (Antinori et al., 2011). The groups of patients considered as LP or LPAD were stratified and analyzed according to this definition.

### Genetic Ambiguities

HIV-1 protease and reverse transcriptase sequences derived from standard genotyping methods were used to determine the recentness of infection, which was calculated based on the ambiguity rate of the genomic sequences. Chronic infection was defined as an ambiguity rate with a cut-off value higher than 0.45% and recent infection as an ambiguity rate with a cut-off value equal to or below 0.45% (Andersson et al., 2013).

### Transmission Cluster Identification

For the TC analysis, the dataset was divided into three separate datasets: subtypes B, A, and G. Control sequences were collected from the Los Alamos database and included all HIV-1 pol subtype (B, A, and G) sequences from Europe, South America, and Africa^1^ (Kuiken et al., 2003). Three reference sequences (from subtypes B and C) were used as outgroup. The resulting dataset was aligned against the global background dataset selected as control using VIRULIGN (Libin et al., 2019). Sequences with low quality, duplicates, and clones were deleted. The sequence dataset for transmission cluster analysis included the sequences of 500 patients from subtypes B, A, and G (obtained from the BEST HOPE dataset) and the sequences included in the control dataset. The total number of sequences used in this analysis was 37,822 (333 seqs were from BEST HOPE and 37,489 seqs were from controls), 7,853 (78 seqs were from BEST HOPE and 7,775 seqs were from controls), and 2,254 (89 seqs were from BEST HOPE and 2,165 seqs were from controls) from subtypes B, A, and G, respectively, with a length of 947 nucleotides. Codon positions associated with drug resistance were removed from the alignment. Maximum likelihood (ML) phylogenies were constructed using FastTree with the generalized time-reversible model. Statistical support of clades was assessed using the Shimodaira-Hasegawa-like test (SH-test). Putative transmission clusters were identified using ClusterPicker v1.332 (Ragonnet-Cronin et al., 2013)[21] and were defined as clades with branch support ≥ 0.99 in the likelihood ratio test (aLRT), as implemented in ClusterPicker v1.332. The clusters were categorized based on the size as large clusters (comprising eight patients or more) and small clusters (comprising less than eight patients). The origin of transmission clusters was assigned if at least 66% of the sequences in the cluster corresponded to the same sampling country. If there was no consistent sampling country for at least 66% of the sequences in the cluster, no origin was assigned.

### Statistical Analysis

Proportions and confidence intervals for proportions were calculated using a 95% Wilson confidence interval for binomially distributed data. The differences between the prevalence of resistance in naïve patients were analyzed using the Mann–Whitney *U* test and the X^2^ tests. Logistic regression was used to examine the association between demographic and clinical factors and the occurrence of SDRMs, and to analyze the trends over time. For all the statistical analyses, we considered a 5% significance level. All the analyses were conducted in SPSS Statistic version 25 software and R3.5.1.

## Results

### Epidemiological and Clinical Data

The characteristics of the study population are presented in Table 1. More than half (77.3%) of the patients (*n* = 820) included in the database were men. The median age at diagnosis was 37.0 (IQR: 29.0–47.0) years. During this period (2014–2019), new diagnoses occurred predominantly (56.0%) between the ages of 22 and 40 years. The main modes of transmission were heterosexual and homosexual contact (49.8% and 47.3%, respectively), followed by intravenous drug use (1.8%). Most patients were born in Portugal (71.9%), and patients born abroad (13.9%) originated from Portuguese-Speaking African Countries (PSAC) and Brazil (11.2%). Individuals with a higher level of education had a higher prevalence when compared to the individuals with other levels of education (35.1%). Most patients (74.4%) were employed; however, 44.2% of them considered the current income as insufficient. More than half (68.5%) were single, 20.9% were married, and 8.3% were divorced and widowers (0.5%). About 75.5% of men reported having sex with men, and 12.9% of men reported having sex with both men and women. However, 100% of women included in the study reported only having sex with men. Based on the percentage of genomic ambiguities, approximately half of the patients (51.3%) presented chronic disease, while 70.0% presented infection stage A. About 82.2% presented no AIDS-defining events, and 70.2% had no sexually transmitted diseases other than HIV. Patients who reported STIs other than HIV were heterosexual (15.3%) and MSMs (43.4%) (data not shown). Patients were predominantly infected with subtype B (40.6%), followed by subtype G (10.9%). At diagnosis, the median viral load (VL) was 4.9 (IQR: 4.4–5.5) Log10 copies/ml, and the CD4 cell count was 339.0 (IQR: 140.0–519.0) cells/μL. About 52.4% of the study population were late presenters. Moreover, the study population presented 31.3% of patients diagnosed as late presenters with advanced disease, with a CD4 count lower than 200 cells/μL. About 77.4% of the LPAD cases were men, and 93.0% were heterosexual. None were MSM. Among these LPAD cases, more than half were born in Portugal (52.8%), and 45.3% were migrants. Among the migrants, 62.5% were born in Africa (data not shown).

**TABLE 1:** Demographic and patients characteristics.

Patient characteristics	Total	With TDR	Without TDR	*p*-value
Total, *n* (%)	820(100%)	89(10.9%)	729(88.9%)	
Gender, *n* (%)	816(99.5%)	90(98.9%)	726(99.6%)	
Male	631(77.3%)	75(83.3%)	556(76.6%)	0.149
Female	185(22.7%)	15(16.7%)	170(23.4%)	
Median age at diagnosis in years IQR, *n* (%)	811(98.9%)	89(97.8%)	722(99.0%)	
	37.0(29.0−	36.0(29.5−	37.0(29.0−	0.651
	47.0)	46.5)	47.0)	
15–21	34(4.2%)	4(4.4%)	30(4.1%)	0.668
22–40	454(56.0%)	54(60.7%)	400(55.4%)	
41–55	231(28.5%)	24(27.0%)	207(28.7%)	
≥ 56	92(11.3%)	7(7.9%)	85(11.8%)	
Transmission route, *n* (%)	811(98.9%)	90(98.9%)	721(98.9%)	
Heterosexual	404(49.8%)	54(60.0%)	350(48.7%)	0.093
MSM**	384(47.3%)	33(36.7%)	351(48.7%)	
IDU	15(1.8%)	1(1.1%)	14(1.9%)	
Other	8(1.0%)	2(2.2%)	6(0.8%)	
Country of origin, *n* (%)	807(98.4%)	91(100%)	716(99.2%)	
Portugal	580(71.9%)	67(73.6%)	513(71.6%)	0.370
Brazil	90(11.2%)	11(12.1%)	79(11.0%)	
Guinea-Bissau	46(5.7%)	2(2.2%)	44(6.1%)	
Angola	34(4.2%)	6(6.6%)	28(3.9%)	
Cabo-Verde	24(3.0%)	1(1.1%)	23(3.2%)	
Mozambique	8(1.0%)	0(0.0%)	8(1.1%)	
Others	25(3.1%)	4(4.4%)	21(2.9%)	
Region of origin, *n* (%)	815(99.4%)	91(100%)	724(99.3%)	
Europe	598(73.4%)	70(76.9%)	528(72.9%)	0.578
Africa	120(14.7%)	9(9.9%)	112(15.3%)	
South America	90(11.0%)	11(12.1%)	79(10.9%)	
Other	7(0.9%)	1(1.1%)	6(0.8%)	
District of residence, *n* (%)	716(87.3%)	81(89.0%)	635(87.1%)	
Lisboa	287(40.1%)	30(37.0%)	257(40.5%)	0.326
Porto	155(21.6%)	17(21.0%)	138(21.7%)	
Faro	82(11.5%)	9(11.1%)	73(11.5%)	
Setúbal	79(11.0%)	6(7.4%)	73(11.5%)	
Aveiro	40(5.6%)	8(9.9%)	32(5.0%)	
Beja	13(1.6%)	2(2.5%)	11(1.7%)	
Coimbra	15(2.1%)	4(4.9%)	11(1.7%)	
Outro	45(6.3%)	5(6.2%)	40(6.3%)	
Migrant status, *n* (%)	815(99.4%)	91(100%)	724(99.3%)	
Migrant	235(28.8%)	24(26.4%)	211(29.1%)	0.582
Native	580(71.2%)	67(73.6%)	513(70.9%)	
School level, *n* (%)	444(54.1%)	39(42.9%)	405(55.6%)	
Third level (9th degree)	98(22.1%)	12(30.8%)	86(21.2%)	
Secondary (12th degree)	143(32.2%)	12(30.8%)	131(32.3%)	0.576
Advanced Technical Specialization	41(9.2%)	2(5.1%)	39(9.6%)	
Higher education (bachelor, master, PhD)	156(35.1%)	13(33.3%)	143(35.3%)	
None	6(1.4%)	0	6(1.5%)	
Current occupation, *n* (%)	433(52.8%)	39(42.9%)	394(54.0%)	
Employed	322(74.4%)	27(69.2%)	295(74.9%)	
Retired	11(2.5%)	1(2.6%)	10(2.5%)	0.033
Sex-worker	1(0.2%)	1(2.6%)	0	
Student	24(5.5%)	2(5.1%)	22(5.6%)	
Unemployed	75(17.3%)	8(20.5%)	67(17.0%)	
Current income, *n* (%)	405(49.4%)	34(37.4%)	371(50.9%)	
Very Insufficient	68(16.8%)	7(20.6%)	61(16.4%)	
Insufficient	179(44.2%)	15(44.1%)	164(44.2%)	0.914
Sufficient	141(34.8%)	11(32.4%)	130(35.0%)	
More than sufficient	17(4.2%)	1(2.9%)	16(4.3%)	
Civil status, *n* (%)	444(54.1%)	40(44.0%)	404(55.4%)	
Single	304(68.5%)	22(55.0%)	282(69.8%)	
Married	93(20.9%)	11(27.5%)	82(20.3%)	0.066
Divorced	37(8.3%)	4(10.0%)	33(8.2%)	
Widower	2(0.5%)	1(2.5%)	1(0.2%)	
Other	8(1.8%)	2(5.0%)	6(1.5%)	
Men sexual partners, *n* (%)	379(60.1%)	35(46.7%)	344(61.9%)	
Men***	286(75.5%)	26(74.3%)	260(75.6%)	0.452
Women	44(11.6%)	6(17.1%)	38(11.0%)	
Men and women	49(12.9%)	3(8.6%)	46(13.4%)	
Women sexual partners, *n* (%)	56(100.0%)	5(100.0%)	51(100.0%)	
Men	56(100%)	5(100.0%)	51(100.0%)	-
Women	0	0	0	
Clinical*				
Type of infection, *n* (%)	820(100%)	91(100%)	729(100%)	
Chronic	421(51.3%)	54(59.3%)	367(50.3%)	0.105
Recent	399(48.7%)	37(40.7%)	362(49.7%)	
Infection stage, *n* (%)	784(95.6%)	86(94.5%)	698(95.7%)	
A	549(70.0%)	51(59.3%)	498(71.3%)	0.034
B	93(11.9%)	11(12.8%)	82(11.7%)	
C	142(18.1%)	24(27.9%)	118(16.9%)	
Aids-defining event, *n* (%)	788(96.1%)	86(94.5%)	702(96.3%)	
Yes	140(17.8%)	23(26.7%)	117(16.7%)	0.021
No	648(82.2%)	63(73.3%)	585(83.3%)	
ISTs, *n* (%)	789(96.2%)	87(95.6%)	702(96.3%)	
Yes	235(29.8%)	27(31.0%)	208(29.6%)	0.787
No	554(70.2%)	60(69.0%)	494(70.4%)	
Subtype, *n* (%)	820(100%)	91(100%)	729(100%)	
HIV-1 Subtype B	333(40.6%)	39(42.9%)	294(40.3%)	0.643
HIV-1 Subtype non-B	487(59.4%)	52(57.1%)	435(59.7%)	
Distribution of subtypes				
HIV-1 Subtype B	333(40.6%)	39(42.9%)	294(40.3%)	0.005
HIV-1 Subtype C	67(8.2%)	18(19.8%)	49(6.7%)	
HIV-1 Subtype G	89(10.9%)	7(7.7%)	82(11.2%)	
HIV-1 Subtype A1	78(9.5%)	6(6.6%)	72(9.9%)	
HIV-1 Subtype D	2(0.2%)	0(0.0%)	2(0.3%)	
HIV-1 Subtype F1	57(7.0%)	4(4.4%)	53(7.3%)	
HIV-1 Subtype H	4(0.5%)	0(0.0%)	4(0.5%)	
HIV-1 CRF-14BG	48(5.9%)	7(7.7%)	41(5.6%)	
HIV-1 CRF-02AG	61(7.4%)	5(5.5%)	56(7.7%)	
HIV-1 recombinants	81(9.9%)	5(5.5%)	76(10.4%)	
Median CD4 count at diagnosis (cells/μL) IQR, *n* (%)	803(97.9%)	90(98.9%)	713(97.8%)	
	339.0(140.0	318.0(73.3	366.3(152.0	0.061
	-519.0)	-513.5)	-522.0)	
< 200	253(31.5%)	36(40.0%)	217(30.4%)	
201-349	168(20.9%)	15(16.7%)	153(21.5%)	0.272
350-500	164(20.4%)	15(16.7%)	149(20.9%)	
> 501	218(27.1%)	24(26.7%)	194(27.2%)	
Late Presentation, *n* (%)	803(97.9%)	90(98.9%)	713(97.8%)	
LP	419(52.2%)	51(56.7%)	368(51.6%)	0.373
NLP	384(47.8%)	39(43.3%)	345(48.4%)	
Viral Load at diagnosis (log10 copies/mL) IQR, n (%)	746(91.0%)	84(92.3%)	662(90.8%)	
	4.95(4.4−	5.0(4.4−	4.94(4.4−	0.310
	5.5)	5.6)	5.5)	
≤ 4.0	122(16.4%)	13(15.5%)	109(16.5%)	
4.1-5.0	291(39.0%)	29(34.5%)	262(39.6%)	0.565
≥ 5.1	333(44.6%)	42(50.0%)	291(44.0%)	

*Clinical type of infection was determined based on the ambiguity rate of genomic sequences. Chronic infection was defined as an ambiguity value > 0.45% and recent infection as an ambiguity value ≤ 0.45% (Andersson et al., 2013). **MSM refers to the transmission route variable, obtained from a closed question in the clinical questionnaires, filled in by the patients’ clinicians (options: “Heterosexual,” “MSM,” “IDU,” and “other”). ***Men sexual partners refer to a question in the sociobehavioral questionnaire, filled in by the patients belonging to the vulnerable groups of migrants, and MSM refers to with whom they usually have sex (options: “I have sex with men,” “I have sex with women,” and “I have sex with men and women”).

### Transmitted HIV Drug Resistance

**FIGURE 1:**
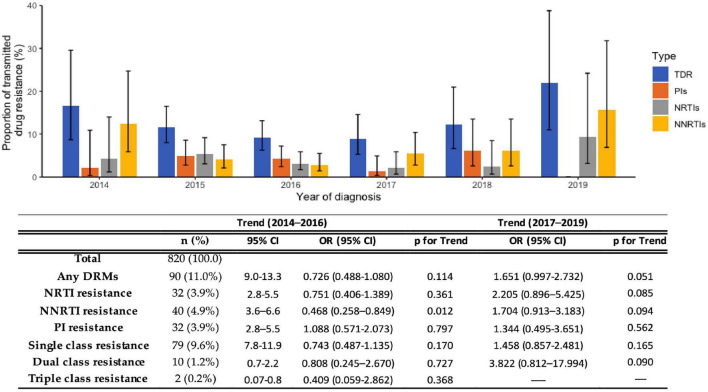
Proportion of transmitted drug resistance (TDR) in sequences obtained from newly diagnosed patients between 2014 and 2019. NRTI, Nucleoside reverse transcriptase inhibitor; NNRTI, Non-nucleoside reverse transcriptase inhibitor; PI, Protease inhibitor; CI, confidence interval; OR, odds ratio.

The overall prevalence of TDR between 2014 and 2019 was 11.0% (95%CI: 9.0–13.3%). Nucleoside reverse transcriptase inhibitor (NRTI) mutations were detected in 3.9% (95%CI: 2.8–5.5%), non-nucleoside reverse transcriptase inhibitor (NNRTI) mutations in 5.0% (95%CI: 3.6–6.6%), and protease inhibitors (PI) in 3.9% (95%CI: 2.8–5.5%) of the HIV cases. In total, 9.6% (95%CI: 7.8–11.9%) presented single-class resistance, 1.2% (95%CI: 0.7–2.2%) dual-class resistance, and 0.2% triple-class resistance (Figure 1). Trends for TDR were determined for the period 2014–2016, where it was decreasing, and for the period 2017–2019, where it was increasing (Figure 1).

Overall TDR presented a decreasing trend from 16.7% in 2014 to 9.2% in 2016 (p_*for–trend*_ = 0.114), and TDR to NRTIs also showed a declining trend (4.17% in 2014 to 3.17% in 2016; p_*for–trend*_ = 0.361). TDR to NNRTIs presented a significantly decreasing trend from 12.5% in 2014 to 2.82% in 2016 (p_*for–trend*_ < 0.05). TDR to PIs, on the other hand, presented an increasing trend (2.08% in 2014 to 4.23% in 2016; p_*for–trend*_ = 0.797). Between 2017 and 2019, TDR presented an increasing trend from 8.9% in 2017 to 21.9% in 2019 (p_*for–trend*_ < 0.05) and also showed an increasing trend for all the drug classes, however, without statistical significance (Figure 1).

**FIGURE 2:**
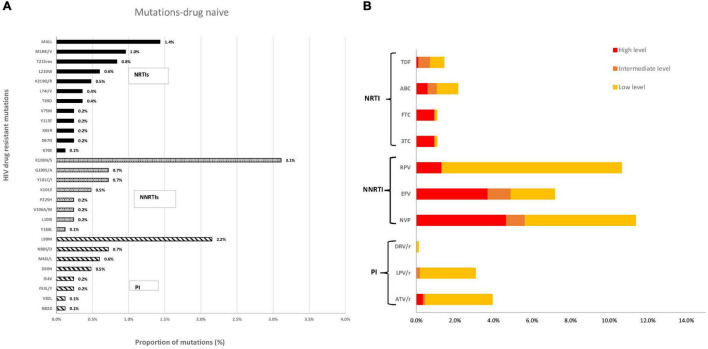
**(A)** Proportion of resistance mutations in the sequences of newly diagnosed patients and **(B)** Predicted phenotypic resistance (Stanford scores) to antiretroviral drugs currently recommended as first-line therapy in Portugal for newly diagnosed patients (2014–2019). NRTI, Nucleoside reverse transcriptase inhibitor; NNRTI, Non-nucleoside reverse transcriptase inhibitor; PI, Protease inhibitor; FTC, Emtricitabine; TDF, Tenofovir; 3TC, Lamivudine; ABC, Abacavir; EFV, Efavirenz; RPV, Rilpivirine; DRV/r, Darunavir; LPV/r, Lopinavir; ATV/r, Atazanavir. Scores of low-level (score 2 and 3), intermediate-level (score 4), or high-level (score 5) resistance were used to predict phenotypic resistance.

According to the HIVdb Stanford database algorithm, NNRTIs presented the highest level of high-level resistance (9.7%) among the drug classes. Nevirapine (NVP) showed the highest proportion of high-level resistance (4.7%), followed by efavirenz (EFV) with 3.7%, and both are related to the most frequently detected mutation K103NS (3.1%). High-level resistance to NRTIs occurred in 2.7% of the patients, with TDR to emtricitabine (FTC) and lamivudine (3TC) presenting the highest levels (1.0%), which is related to M41L (1.4%) mutation. High-level resistance to PIs was found in 0.4% of the patients, with atazanavir (ATV) presenting 0.4% of high-level resistance, which is related to the presence of L90M mutation (2.2%) (Figures 2A,B).

We also analyzed the association between HIV drug resistance mutations and subtypes of infection. We observed that individuals infected with subtype B were more likely to develop mutations associated with resistance to all the antiretroviral classes (NRTIs, NNRTIs, and PIs). In the individuals infected with subtype B, resistance to NRTIs can be attributed mainly to the M41L (0.8%), K219QR, and T215rev (0.4%) mutations. For NNRTIs, the most common mutations were K103NS (1.20%) and G190SA (0.4%), and for PIs, the main mutation found was M46IL (0.6%) (data not shown).

### Predictors of Transmitted Drug Resistance

The clinical and sociodemographic factors significantly associated with TDR in the univariate model were transmission route (sex between men, OR = 0.62), stage of infection (stage C, OR = 2.04), having an AIDS-defining event (patients without AIDS-defining events, OR = 0.52), and being infected with subtype C (OR = 2.86). The multivariate analysis indicated that TDR was significantly associated with the transmission route (MSM presented a lower probability of having TDR when compared to the heterosexual contact) and infection with subtype C (compared to subtype B) (Table 2).

**TABLE 2:** Unadjusted and adjusted regression analysis of factors associated with HIV-transmitted drug resistance.

Any TDR		Unadjusted		Final model	
		OR (95%CI)	*p*-value	aOR (95%CI)	*p*-value
Sex, *n* (%)	Male	1	1	1	1
	Female	1.53 (0.86–2.74)	0.149	1.75 (0.89–3.44)	0.10
Age groups	15–21	1	1		
	25–40	1.06 (0.36–3.11)	0.92		
	41–55	0.91 (0.30–2.81)	0.88		
	≥56	0.64 (0.18–2.32)	0.49		
Transmission route	Heterosexual	1	1	1	1
	MSM	0.62 (0.40–0.98)	0.04	0.55 (0.30–1.02)	0.06
	IDU	0.48 (0.06–3.72)	0.48	0.00	1
	Other	2.24 (0.44–11.36)	0.33	1.51 (0.22–10.5)	0.68
Country of origin	Portugal	1	1		
	Brazil	1.08 (0.55–2.13)	0.82		
	Guinea-Bissau	0,36 (0,09–1.51)	0.16		
	Angola	1.69 (0.67–4.21)	0.26		
	Cabo-Verde	0.33 (0.04–2.46)	0.28		
	Mozambique	0.00	1.00		
	Others	1.50 (0.50–4.50)	0.47		
Region of origin	Europe	1			
	Africa	0.06 (0.54–2.10)	0.86		
	South America	0.62 (0.30–1.28)	0.20		
	Other	1.29 (0.15–10.88)	0.82		
District of residence	Lisboa	1			
	Porto	1.04 (0.56–1.96)	0.89		
	Faro	1.04 (0.47–2.29)	0.92		
	Setúbal	0.69 (0.28–1.73)	0.43		
	Aveiro	2.10 (0.89–4.96)	0.09		
	Beja	1.58 (0.33–7.45)	0.57		
	Coimbra	1.06 (0.40–2.88)	0.91		
	Outro	2.89 (0.88–9.52)	0.08		
Migrant status	Migrant	1			
	Native	0.89 (0.54–1.45)	0.63		
School level	Third level (9th degree)	1			
	Secondary (12th degree)	0.65 (0.28–1.52)	0.32		
	Advanced Technical Specialization	0.37 (0.08–1.48)	0.21		
	Higher education (bachelor, master, PhD)	0.65 (0.28–1.48)	0.30		
	None	0	0.99		
Current occupation	Employed	1			
	Retired	1.10 (0.14–9.0)	0.93		
	Sex-worker		1		
	Student	0.96 (0.21–4.29)	0.96		
	Unemployed	1.30 (0.57–2.98)	0.54		
Current income	Very Insufficient	1			
	Insufficient	0.81 (0.32–2.09)	0.67		
	Sufficient	0.76 (0.28–2.04)	0.58		
	More than sufficient	0.56 (0.06–4.91)	0.60		
Civil status	Single	1			
	Married	1.73 (0.80–3.71)	0.16		
	Divorced	1.58 (0.51–4.87)	0.42		
	Widower	13.04 (0.79–215.7)	0.07		
	Other	4.35 (0.83–22.8)	0.08		
Men sexual partners, *n* (%)	Men	1			
	Women	1.58 (0.61–4.08)	0.35		
	Men and women	0.65 (0.19–2.24)	0.50		
Type of infection	Chronic	1			
	Recent	0.68 (0.44–1.06)	0.092		
Infection stage	A	1		1	
	B	1.31 (0.66–2.62)	0.44	1.16 (0.51–2.65)	0.72
	C	2.04 (1.21–3.45)	0.01	1.67 (0.38–7.40)	0.50
Aids-defining event	Yes	1		1	1
	No	0.53 (0.32–0.89)	0.02	0.76 (0.17–3.38)	0.72
ISTs	Yes	1			
	No	1.07 (0.66–1.73)	0.79		
Subtype	HIV-1 Subtype B	1			
	HIV-1 Subtype non-B	1.09 (0.70–1.69)	0.71		
Distribution of Subtypes	HIV-1 Subtype B	1		1	
	HIV-1 Subtype C	2.86 (1.52–5.40)	0.001	3.10 (1.50–6.44)	0.002
	HIV-1 Subtype G	0.63 (0.27–1.47)	0.29	0.62 (0.25–1.55)	0.31
	HIV-1 Subtype A1	0.63 (0.26–1.55)	0.32	0.77 (0.30–1.98)	0.59
	HIV-1 Subtype D	0.00	1.0	0.00	1
	HIV-1 Subtype F1	0.59 (0.20–1.71)	0.33	0.59 (0.19–1.85)	0.29
	HIV-1 Subtype H	0.00	1.0	1.06 (0.38–2.94)	0.91
	HIV-1 CRF-14BG	0.70 (0.26–1.84)	0.47	0.58 (0.19–1.85)	0.36
	HIV-1 CRF-02AG	1.33 (0.56–3.17)	0.52	1.06 (0.38–2.94)	0.91
	HIV-1 recombinants	0.51 (0.20–1.34)	0.18	0.39 (0.13–1.20)	0.10
CD4 count at diagnosis (cells/μL)	< 200	1			
	201–350	0.59 (0.31–1.12)	0.11		
	351–500	0.61 (0.32–1.15)	0.13		
	> 501	0.75 (0.43–1.30)	0.30		
Late presentation	LP	1			
	NLP	0.82 (0.52–1.27)	0.37		
Viral load at diagnosis (log10 copies/mL)	≤4.0	1			
	4.1–5.0	0.93 (0.47–1.86)	0.84		
	≥5.1	1.19 (0.62–2.31)	0.60		

### Inference of Transmission Clusters

Based on the PR+RT phylogenetic analysis, transmission clusters (TCs) were defined as clades with a branch support value *≥* 99% for subtypes B, A, and G.

We identified 87 transmission clusters comprising 273 of the 500 patients (54.6%). The average cluster size was 17.4, with a minimum of 2 (31 clusters) and a maximum of 118 (1 cluster).

When the proportion of transmission clusters between the subtypes was compared, patients carrying subtype G strains were more likely to be inside the clusters (67/89; 75.3%), followed by subtypes B (180/333; 54.1%) and A (22/78; 28.2%) (*p* < 0.001). Subtype G and A sequences in clusters presented a high proportion of heterosexual patients (84.1% and 57.1%, respectively). On the other hand, among subtype B sequences inside the clusters, 72.7% belonged to the MSM population.

Among the subtypes B, A, and G, 52 out of 500 (10.6%) patients presented HIV drug resistance, while almost the same proportion was observed in transmission clusters (9.5%). The highest prevalence of TDR (73%) was observed among small clusters (cluster size lower than 10 sequences). When we analyzed TDR inside the clusters between the subtypes, we observed different proportions of TDR. Subtypes A1 (13.6%) and B (9.8%) were more likely to carry SDRMs inside the clusters when compared to subtype G (7.5%). Regardless of the subtypes, the heterosexual population presented the highest proportion of TDR compared to MSMs. Among subtype A1, heterosexuals presented 16.7% of TDR, followed by 14.3% in patients infected with subtype B and in lower proportion (9.4%) in patients with subtype G (Tables 3, 4).

**TABLE 3:** Subtypes, TDR, and risk factor of patients associated with HIV-1 molecular transmission clusters.

	Cluster *N* = 273	Non-cluster *N* = 227	*p* value
Subtypes			< 0.0001
A	28.2(22/78)	71.8(56/78)	
B	55.3(180/333)	44.7(149/333)	
G	75.3(67/89)	24.7(22/89)	
**TDR**			
Overall	9.5(26/273)	11.5(26/227)	0.730
A	13.6(3/22)	5.4(3/56)	0.192
B	9.8(18/184)	14.0(21/149)	0.235
G	7.5(5/67)	9(2/22)	0.821
**Risk factor**			0.219
MSM	53.0(150/283)	47.0(133/283)	
Heterosexual	57.0(114/200)	43.0(86/200)	

**TABLE 4:** Characteristic of patients in HIV-1 molecular transmission clusters according to the transmission route.

	MSM	Heterosexual	*p* value
Subtypes			< 0.0001
A (*n* = 21)	42.9(9/21)	57.1(12/21)	
B (*n* = 180)	72.7(131/180)	27.3(49/180)	
G (*n* = 63)	15.9(10/63)	84.1(53/63)	
TDR			
Overall (264)	9.3(14/150)	9.6(11/114)	0.900
A (*n* = 21)	11.1(1/9)	16.7(2/12)	0.699
B (*n* = 180)	7.6(10/131)	14.3(7/49)	0.189
G (*n* = 63)	0(0/53)	50.0(5/10)	–

## Discussion

This study aimed to understand HIV-1-transmitted drug resistance in newly diagnosed patients by providing a current picture of transmitted drug resistance patterns in these populations. This study is particularly important, as it provides information that can guide the development of preventive measures directed at specific risk populations.

The study population was mostly composed of men (77.3%). The most prevalent age group was 22–40 years, and most patients originated from Portugal and were included in the heterosexual and MSM risk groups. The characteristics of this population are consistent with the patterns reported in the latest Portuguese health authorities report. Clinically, it is worth emphasizing the high prevalence of very late presenters (LPAD; 31.5%) identified in this study, which is also consistent with the prevalence reported in the Portuguese health authorities report (Direção Geral da Saúde and Instituto Nacional de Saúde Doutor Ricardo Jorge, 2020).

Our study showed that the estimated prevalence of TDR in Portugal was 11.0% (IC95%: 9.0–13.3) in patients diagnosed between 2014 and 2019. A decreasing trend for TDR was also observed between 2014 and 2016. A similar trend was observed for NRTIs and NNRTIs, but not for PIs, which showed increasing TDR in this period. Between 2017 and 2019, an increasing trend was observed for TDR and all drug classes. This increasing trend in TDR had already been observed by our study group in a longitudinal study that included patients between 2001 and 2017 (Pingarilho et al., 2020), as well as in a study published by a Canadian group (Rocheleau et al., 2018). This increase could be related to population mobility and an increase in the number of migrants from Portuguese-speaking Sub-Saharan African countries, where TDR has been increasing in the last few years (Rhee et al., 2015; Pingarilho et al., 2018; Sebastião et al., 2019; Pimentel et al., 2020).

The most prevalent mutation detected was K103N, which confers high-level resistance to NVP and EFV, followed by M41L, which reduces susceptibility to TDF and ABC when in combination with other NRTI mutations, and M184VI, which causes high-level resistance to 3TC and FTC. L90M resistance mutation presented the highest prevalence for PIs, and it causes reduced susceptibility to ATV and LPV. L90M mutation (1.8%) was widely observed in patients infected with subtype C, and this could be the reason for the increased resistance to PIs noticed in our study (3.9%), compared to 2.8% obtained in our previously published study (Pingarilho et al., 2020).

We also observed that the risk of TDR was significantly higher in patients infected with subtype C when compared to those infected with subtype B. We hypothesize that this finding could be explained by the higher prevalence of L90M mutations among MSMs infected with subtype C, eventually caused by its forward transmission in MSM transmission clusters. However, in this study, the transmission clusters of subtype C were not reconstructed, since the prevalence of this subtype was lower than 10%. Future studies will address this problem.

Although the L90M mutation was more frequent among the MSM group, in fact, the overall TDR was 1.7 times (*p* = 0.027) higher within the heterosexual population than that observed in the MSM group. This result is discordant from previous studies that have shown faster onward transmission of HIV infection with less reversion of DRM in the MSM transmission clusters and therefore a potentization of the transmission of TDR (Vercauteren et al., 2009). However, this finding agrees with the results obtained through transmission cluster analyses, which showed that subtype G heterosexuals (Pineda-Peña et al., 2019) were more frequently inside clusters when compared to subtypes A and B, indicating that transmission is more active in this subgroup. This is in contrast to what other European studies showed, where it was concluded that non-B subtypes are associated with the heterosexual population and are less frequently found in the transmission clusters (Lorenzin et al., 2019; Paraskevis et al., 2019; Pimentel et al., 2022). Since our study is based on newly diagnosed patients, we believe that our results present a more recent view of the epidemic in the country that could already reflect a successful impact of pre-exposure prophylaxis (PrEP) among the MSM patients, with a slowdown of HIV transmission clusters in this risk group. We hypothesize that MSM patients, in addition to auto-testing more frequently, have a higher risk perception that plays an important role in the acceptance of PrEP (Plotzker et al., 2017). Consistently with our hypothesis, some other studies have already reported that there is a high level of willingness and acceptance of PrEP use among MSM (Frankis et al., 2016; Phan and Vu, 2017; Spinner et al., 2018; Nguyen et al., 2021). Moreover, in Portugal, it is known that almost the entire population interested in using PrEP is MSM; however, no such study has been published until now. Nevertheless, a study conducted in 2016 reported that in France, more than 95% of the people interested in the use of PrEP were MSM (Loos et al., 2016).

The overall rate of TDR was 9.5% inside the clusters compared to 11.5% outside the clusters, and the highest proportion of TDR was observed in small clusters (73%), mostly composed of heterosexuals. Subtype A presented the highest prevalence of TDR in clusters, followed by subtypes B and G. However, consistent with the results of the analyses of TCs, we observed that heterosexual individuals from subtype G presented higher levels of TDR in clusters, compared to MSMs of the same subtype. The same was observed for subtypes A and B, where heterosexuals inside the clusters presented higher levels of TDR compared to MSM, despite the fact that subtype B presented a higher rate of MSM. Heterosexual transmission accounts for approximately 57.8% of the new HIV infections in Portugal in the last few years, with migrants contributing to approximately 43.1% of these new infections, mostly from Sub-Saharan African countries (51.2%), where heterosexual transmission is predominant (Direção Geral da Saúde and Instituto Nacional de Saúde Doutor Ricardo Jorge, 2020). Most of these heterosexual contacts present small clusters of two individuals (men–women), indicating low levels of forward transmission that still represent a high proportion of TDR transmission in Portugal.

Given the new trends presented in this manuscript, our results seem to indicate that the successful HIV prevention measures implemented in the MSM populations, which include the use of PreP (pre-exposure prophylaxis), frequent medical appointments and testing, and earlier diagnosis in community-based centers, seem to have been successful in decreasing the HIV transmission and consequently the TDR among the MSM group. Since, in Portugal, PreP is mostly used by the MSM population and not by heterosexuals, this can explain the higher proportion of heterosexuals in TCs and hence higher TDR transmission in heterosexuals. Also, it is important to note that the MSM population is more frequently tested and has an opportunity for earlier diagnosis, which may imply less transmission of HIV and thus TDR. HIV-1 prevention measures should now be strengthened for heterosexual risk groups.

## Conclusion

The transmission patterns of HIV-1 are changing in Portugal, probably due to the new prevention measures introduced in the country and which are mostly accepted by the MSM groups. However, it is very important to address the heterosexual group where TDR is increasing and which presents high levels of late diagnosis. For this reason, it is important to develop preventive measures for HIV-1 transmission addressing the specificities of this group.

## Data Availability Statement

The data analyzed in this study is subject to the following licenses/restrictions: The dataset would be available through requisition and explanation of the study purposes. Requests to access these datasets should be directed to AS, ana.abecasis@ihmt.unl.

## Other members of the BESTHOPE study group

Ana Bandeiras, Ana Pimenta, Anabela Granado, André Gomes, António Maio, Catarina Messias, Celina Bredes, Diana Seixas, Diva Trigo, Edite Mateus, Fátima Gonçalves, Filipa Azevedo, Francisco Vale, Henriqueta Pereira, Inês Siva, Isabel Casella, Isabel Diogo, Isabel Neves, Joana Sá, Joana Simões, Joana Granado, Joana Vasconcelos, João Cabo, João Pereira-Vaz, João Domingos, João Torres, Joaquim Cabanas, Johana Jesus, José Melo Cristino, Karen Pereira, Luís Caldeira^†^, Luísa Sêco, Lurdes Correia, Manuela Simão, Maria Saudade Ivo, Mariana Pessanha, Marta Feijó, Margarida Cardoso, Nildelema Malaba, Nádia Gomes, Natália Patrício, Nuno Luís, Nuno Janeiro, Patrícia Carvalho, Paula Brito, Pedro Simões, Rosário Prazos, Sara Lino, Sara Casanova, Sofia Pinheiro, Sónia Marques, Sofia Jordão, Sueila Martins, Telma Azevedo, Teresa Meira, Vanda Mota, and Vanda Silva.

## Author Contributions

MP and AA: conceptualization and writing—original draft and writing—reviewing and editing. MP, MNM, and VP: data curation and validation. MP, MNM, VP, and AA: formal analysis. AA: funding acquisition, project administration, and supervision. MP, VP, PG, and MRM: investigation. MP, VP, MRM, and AA: methodology. PG, AS, AD, BA, CP, CK, CR, CC, CM, DF, ES, ET, FMo, FaR, FMa, FeR, GG, HR, IC, IG, JSi, JO, JF, JP, JC, JSo, JH, KM, LP, MA, MG, MJM, MGM, MS, MC, NM, OC, PPa, PPr, PR, RP, RT, RA, RR, RS, RC, SN, TF, and TB: resources, data collection, and approval of the publication contents. All authors contributed to the article and approved the submitted version.

## Conflict of Interest

The authors declare that the research was conducted in the absence of any commercial or financial relationships that could be construed as a potential conflict of interest.

## Publisher’s Note

All claims expressed in this article are solely those of the authors and do not necessarily represent those of their affiliated organizations, or those of the publisher, the editors and the reviewers. Any product that may be evaluated in this article, or claim that may be made by its manufacturer, is not guaranteed or endorsed by the publisher.

## Footnotes

1
http://www.hiv.lanl.gov
